# Early outcome of endoscopic mitral valve surgery in elderly patients: a high-volume single center experience

**DOI:** 10.3389/fcvm.2023.1182752

**Published:** 2023-11-28

**Authors:** Jonas Pausch, Oliver D. Bhadra, Xiaoqin Hua, Philipp Stolfa, Carolin Kuhlmann, Mirko Voß, Evaldas Girdauskas, Hermann Reichenspurner, Lenard Conradi

**Affiliations:** ^1^ Department of Cardiovascular Surgery, University Heart and Vascular Center Hamburg, Hamburg, Germany; ^2^ Department of Cardiovascular and Thoracic Surgery, University Hospital Augsburg, Augsburg, Germany

**Keywords:** mitral regurgitation, minimally-invasive mitral valve surgery, endoscopic mitral valve surgery, mitral valve repair, transcatheter mitral valve repair

## Abstract

**Introduction:**

Despite increasing use of transcatheter approaches, endoscopic mitral valve surgery (MVS) remains an established option for treatment of mitral regurgitation (MR). Nevertheless, as perioperative risk increases with age, outcome of endoscopic MVS in elderly patients is uncertain.

**Methods:**

We retrospectively analyzed 756 consecutive patients with MR ≥2, who underwent minimally-invasive MVS at our institution between 2016 and 2022. Patients were stratified by age ≥75 (elderly-group; *n *= 91) or <75 years (control-group; *n *= 665). All patients received endoscopic MVS via right anterolateral minithoracotomy with non-rib spreading soft tissue retraction and 3D-camera visualization.

**Results:**

Overall surgical risk was increased in the elderly-group (median age of 77 (76–80) years vs. 58 (51–67) years, *p* < 0.001) with STS-PROM Scores of 1.9% vs. 0.4% (*p* < 0.001) and increased prevalence of hypertension, diabetes, coronary artery disease and atrial fibrillation (AFib). Elderly patients were also more symptomatic (*N*YHA class III 45.7% vs. 29.8%; *p *= 0.002). Axillo-femoral perfusion was more frequently used in the elderly-group (27.5% vs. 4.2%; *p* < 0.001). Cross-clamp and cardiopulmonary bypass times were similar. Rate of MV repair was 85.7% vs. 93.8% (*p *= 0.005). Closure of the left atrial appendage was more frequently performed in the elderly-group (45.1% vs. 23.9%; *p* < 0.001), whereas rate of concomitant tricuspid valve repair was similar (11.0% vs. 8.9%; *p* = 0.511). Postoperative complications including perioperative hemodialysis (3.3% vs. 2.9%; *p* = 0.739), low cardiac output (5.3% vs. 3.8%; *p* = 0.393), perioperative stroke (1.1% vs. 0.15%; *p* = 0.224) and myocardial infarction (0% vs. 0.15%) were favorably low in both groups. Acute mortality at 30 days was 2.2% vs. 0.4% (*p* = 0.112).

**Conclusion:**

Despite increased prevalence of outcome-relevant comorbidities and surgical risk, perioperative outcome of patients aged ≥75 years undergoing endoscopic MVS is favorable. Therefore, endoscopic MVS is a valuable therapeutic option for selected elderly patients and should be taken in consideration during routine heart-team discussion.

## Introduction

Prevalence of MR, which represents the second most common type of valvular heart disease (VHD) in Europe, increases with age (1). Moderate to severe MR is present in approximately 8% of the population aged ≥75 years, and about half of cases remain undiagnosed and thus untreated (2), resulting in increased morbidity and mortality. In addition to conventional MVS, which still is the most prevalent strategy to address MR (3), transcatheter mitral valve (MV) technologies developed as a viable treatment option for high-risk (4) and/or elderly patients (5). Despite promising periprocedural results, long-term durability of transcatheter edge-to-edge repair (TEER) remains inferior compared to conventional MVS, in particular in the treatment of primary MR (PMR) (4, 5).

**Figure 1:**
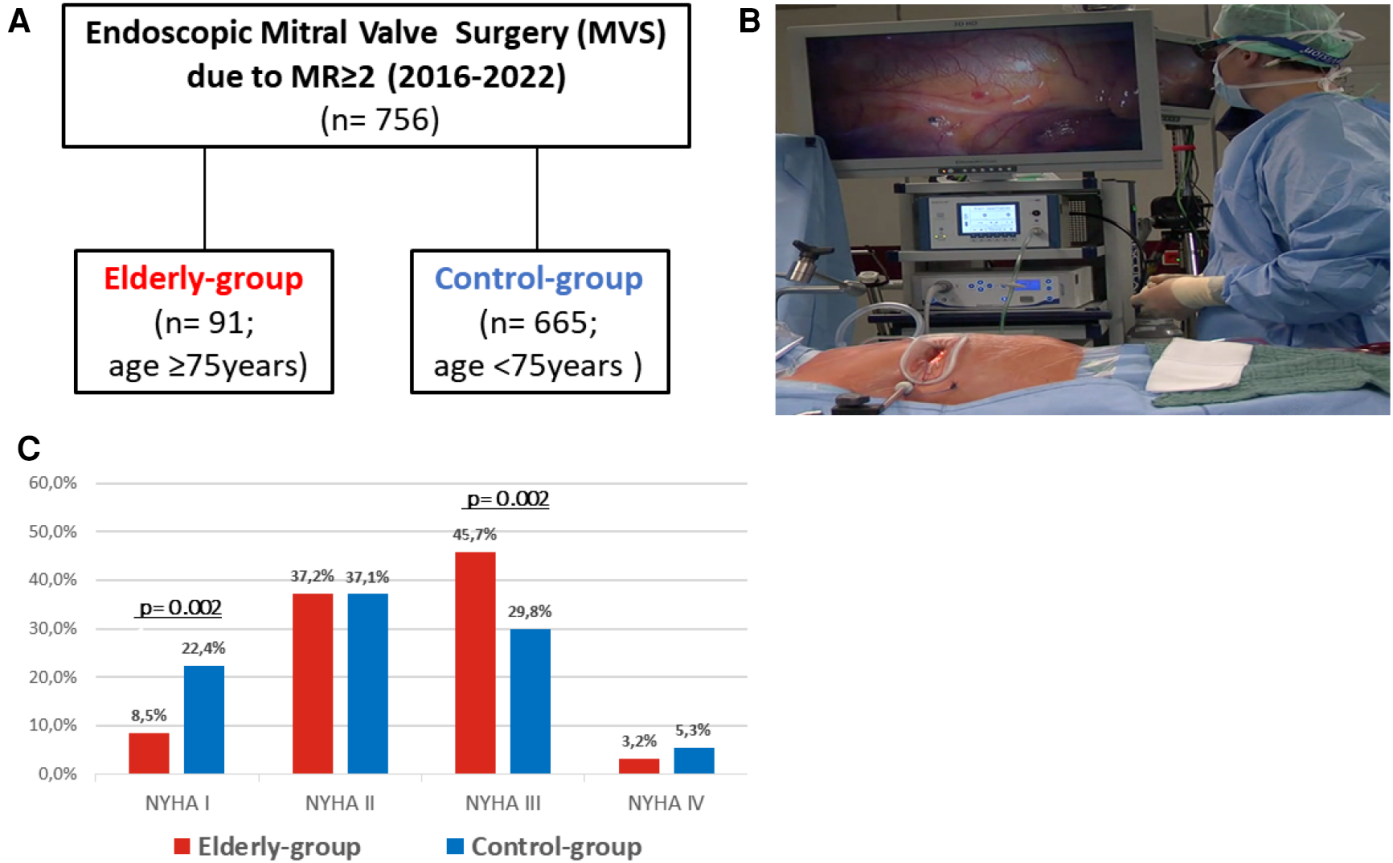
Study design (**A**), surgical set-up (**B**) and prevalence of NYHA class symtoms (**C**).

Minimally-invasive MVS via anterolateral mini-thoracotomy evolved as a safe alternative to conventional sternotomy (6), resulting in lower postoperative ventilation times, transfusion rates, shorter stay on intensive care unit (ICU) and shorter in-hospital stay (7). In particular elderly, frail patients may potentially benefit from reduced surgical trauma. Nevertheless, due to an increased prevalence of outcome-relevant comorbidities and elevated perioperative risk (8), outcome of minimally-invasive MVS in elderly patients remains uncertain (9). Therefore, the aim of our study was to analyze perioperative outcome of consecutive patients aged ≥75 years undergoing minimally-invasive endoscopic MVS due to MR≥2 at our institution between 2016 and 2022 (Figure 1A).

## Patients and methods

### Ethical statement

Due to anonymous data collection and retrospective study-design, informed patient consent was waived according to a statement of our local ethics committee. The study complies with the Declaration of Helsinki.

### Patients

We herein report periprocedural outcome of 756 consecutive patients undergoing endoscopic MVS due to relevant MR at our institution between 2016 and 2022. Patients were stratified according to age ≥75 (elderly-group; *n* = 91) or <75 years (control-group; *n* = 665) (Figure 1A).

### Inclusion criteria

All 756 consecutive patients undergoing endoscopic MVS at our institution between 2016 and 2022, were retrospectively analyzed. Patients receiving MVS via full-sternotomy, mainly due to concomitant CABG or aortic valve surgery were excluded from analysis. Furthermore, patients with severe MV stenosis and acute infective endocarditis were excluded.

### Surgical setup and technique of endoscopic mitral valve surgery

Endoscopic MVS was performed via right anterolateral mini-thoracotomy under 3D endoscopic guidance. A limited skin incision (<4 cm) was used to enter the fourth intercostal space. A non-rib spreading soft tissue retractor was placed to facilitate appropriate surgical access (Figure 1B). Peripheral cardiopulmonary bypass (CPB) was established via percutaneous cannulation of the femoral artery or open direct cannulation of femoral or axillary arteries. Venous cannulation was performed using the right femoral vein either via surgical cut-down and direct visualization or percutaneously. In patients >85 kg or in patients requiring concomitant tricuspid valve repair, a second venous cannula was inserted percutaneously via the right jugular vein to optimize venous drainage during cardiopulmonary bypass (CPB) or to prepare for total bypass. Aortic occlusion was performed using a transthoracic aortic clamp and antegrade cardioplegia was administered via the aortic root. After exposure of the MV using direct left atrial access, standardized intraoperative examination was performed to determine the etiology of MR and define the surgical strategy. At the surgeon`s discretion, MV repair or replacement was performed using the appropriate surgical techniques (e.g., chordal replacement, leaflet resection, Alfieri stich). Concomitant procedures such as atrial fibrillation (AFib) ablation, left atrial appendage (LAA) closure or tricuspid valve repair were performed according to our institutional standards.

### Statistical analysis

Anonymous retrospective data collection was obtained using a specifically designed MVS-database. Normally distributed continuous variables are presented as mean values and standard deviation, whereas median and interquartile range are used for non-normally distributed continuous variables. Absolute numbers and percentages are used for categorical variables. Unpaired *t*-test was used for between-group comparison of normally distributed numeric variables, otherwise Mann–Whitney *U*-test was used. *χ*^2^ test was performed for between-group comparison of categorial variables if the minimum expected cell size was at least 5. Otherwise, Fisher's exact test was used. Kaplan–Meier analysis and logrank test was used to compare mid-term survival during. If *p*-value was <0.05, results were considered statistically significant. IBM Corp. Released 2019. IBM SPSS Statistics for Windows, Version 26.0. Armonk, NY: IBM Corp. was used for all statistical analyzes. The data underlying this article will be shared on reasonable request to the corresponding author.

## Results

### Study population

Between 2016 and 2022, a total of 756 consecutive patients underwent endoscopic MVS at our institution due to MR ≥ 2 and were stratified according to age ≥75 or <75 years at the time of surgery (Figure 1A) resulting in median age of 77 (76–80) vs. 58 (51–67) years (*p* < 0.001) in groups respectively. In addition, prevalence of outcome-relevant comorbidities including hypertension, diabetes, coronary artery disease or AFib was increased within the elderly-group (Table 1). Of note, patients in the elderly-group, were more likely to present with NYHA class III symptoms (45.7% vs. 29.8%; *p* = 0.002) (Figure 1C) and increased baseline serum-levels of creatinine and natriuretic peptide. According to standard operative risk stratification, patients in the elderly group presented with an overall increased surgical risk profile (STS PROM: 1.9% vs. 0.4%; *p* < 0.001) (Table 1).

**Table 1:** Preoperative patient characteristics and echocardiographic parameters.

Variables	Elderly group (*n* = 91)	Control group (*n* = 665)	*p*-value
Age (years), median (IQR)	77 (76–80)	58 (51–67)	<0.001
Male, *n* (%)	49 (53.8)	438 (64.1)	0.059
BMI (kg/m^2^), median (IQR)	24.4 (22.1–27.7)	24.6 (22.2–27.4)	0.756
s/p malignancy, *n* (%)	10 (11.0)	48 (7.2)	0.208
s/p stroke, *n* (%)	6 (6.6)	29 (4.4)	0.299
COPD, *n* (%)	4 (4.4)	21 (3.2)	0.529
Diabetes mellitus, *n* (%)	10 (11.0)	30 (4.5)	0.015
Art. Hypertension, *n* (%)	56 (61.5)	277 (41.7)	0.002
Atrial fibrillation, *n* (%)	52 (57.1)	193 (29.0)	<0.001
Coronary artery disease, *n* (%)	25 (27.4)	94 (14.1)	<0.001
STS-Mortality Score %, median (IQR)	1.9 (1.6–3.3)	0.38 (0.26–0.60)	<0.001
Hemoglobin (g/dl),	13.2 (12.2–14.0)	14.1 (13.0–14.8)	<0.001
Creatinin, mg/dl, median (IQR)	1.0 (0.9–1.3)	0.91 (0.8–1.1)	<0.001
NT-proBNP (pg/dl), median (IQR)	1,230 (392–2,766)	354 (128–1,354)	<0.001
Primary MR, *n* (%)	63 (69.2)	517 (77.7)	0.052
Secondary MR, *n* (%)	22 (24.2)	87 (13.1)	0.003
LVEF (%), mean ± SD	57.6 ± 9.6	59.0 ± 9.7	0.228
TAPSE (mm), mean ± SD	23.0 ± 5.3	24.6 ± 5.6	0.021
Tricuspid regurgitation (TR) ≥ 2, *n* (%)	37 (40.7)	127 (19.1)	0.003
Systolic pulmonary artery pressure (mmHg), mean ± SD	50.0 ± 13.6	43.6 ± 13.9	<0.001
LA-Volume (ml), median (IQR)	95 (78–113)	94 (73–123)	0.858

SD, standard deviation; BMI, body mass index; COPD, chronic obstructive pulmonary disease; GOLD, global initiative for chronic obstructive lung disease; NT-pro-BNP, N-terminal pro-B natriuretic peptide; IQR, interquartile range; LVEF, left ventricular ejection fraction; LVEDD, left ventricular end-diastolic diameter; LA-volume, left atrial volume; TAPSE, tricuspid annular plane systolic excursion; sPAP, systolic pulmonary artery pressure; s/p, status post.

### Preoperative echocardiographic characteristics

All patients within both groups, preoperatively presented with MR ≥ 2. The rate of concomitant relevant tricuspid regurgitation (TR) ≥2 was increased in the elderly-group (40.7% vs. 19.1%; *p* = 0.003). In contrast to comparable rates of PMR, which was present in 69.2% (elderly-group) vs. 77.7% (control-group) of patients (*p* = 0.052), prevalence of secondary MR (SMR) was significantly increased in the elderly-group (24.2% vs. 13.1%; *p* = 0.003). Prolapse of the posterior mitral leaflet (PML) represented the most common cause of PMR and was similarly prevalent in groups (42.2% vs. 47.8%; *p* = 0.316). Left ventricular ejection fraction (LVEF) was similar in both groups (*p* = 0.228), whereas right ventricular (RV) function, measured as tricuspid annular plane systolic excursion (TAPSE), was significantly impaired in the elderly-group in comparison to the control group (23.0 ± 5.3 vs. 24.6 ± 5.6; *p* = 0.003). Furthermore, systolic pulmonary artery pressure was increased within the elderly patients (Table 1).

### Procedural data

Surgical details are summarized in Table 2. Briefly, direct arterial cannulation of the axillary artery was more frequently used within elderly vs. control group (27.5% vs. 4.2%; *p* < 0.001). MV repair was performed in 85.7% vs. 93.8% of patients (*p* = 0.055). Median annuloplasty ring size used, was 32 (30–34) vs. 34 (30–36) mm (*p* < 0.001). Implantation of neochords (51.6% vs. 69.5%; *p* = 0.261), as well as leaflet resection (23.1% vs. 14.7%; *p* = 0.136) were predominantly used to correct leaflet prolapse or flail. Concomitant occlusion of the LAA was performed in 45.1% vs. 23.9% of patients (*p* < 0.001). Cross-clamp and cardiopulmonary bypass times were similar between groups, whereas procedural time was increased within the elderly group (Table 2).

**Table 2:** Intraprocedural characteristics.

Variables	Elderly group (*n* = 91)	Control group (*n* = 665)	*p*-value
MV-repair, *n* (%)	78 (85.7)	624 (93.8)	0.005
MV-replacement, *n* (%)	13 (14.3)	41 (6.2)	0.005
MV ring size (mm), median (IQR)	32 (30–34)	34 (30–36)	<0.001
Neochord implantation, *n* (%)	47 (51.6)	462 (69.5)	0.261
Leaflet resection, *n* (%)	21 (23.1)	98 (14.7)	0.136
Alfieri-stich, *n* (%)	6 (6.6)	37 (5.6)	0.615
Left atrial appendage occlusion, *n* (%)	41 (45.1)	159 (23.9)	<0.001
Concomitant ablation of AFib, *n* (%)	27 (29.7)	134 (20.1)	0.034
Concomitant TV-repair, *n* (%)	10 (11.0)	59 (8.9)	0.511
Axillo-femoral cannulation, *n* (%)	25 (27.5)	28 (4.2)	<0.001
Cross-clamp time (min), median (IQR)	84 (73–110)	92 (76–113)	0.182
Cardiopulmonary bypass time (min), median (IQR)	158 (135–186)	158 (134–190)	0.698
Procedural time (min), median (IQR)	250 (205–291)	228 (186–273)	0.010
Perioperative ECLS-support, *n* (%)	0 (0.0)	7 (1.1)	1.0
Perioperative IABP-support, *n* (%)	0 (0.0)	2 (0.3)	1.0

MV, mitral valve; AFib, atrial fibrillation; TV, tricuspid valve; ECLS, extracorporeal life support; IABP, intra-aortic balloon pump.

### Perioperative results and 30-day mortality

**Figure 2:**
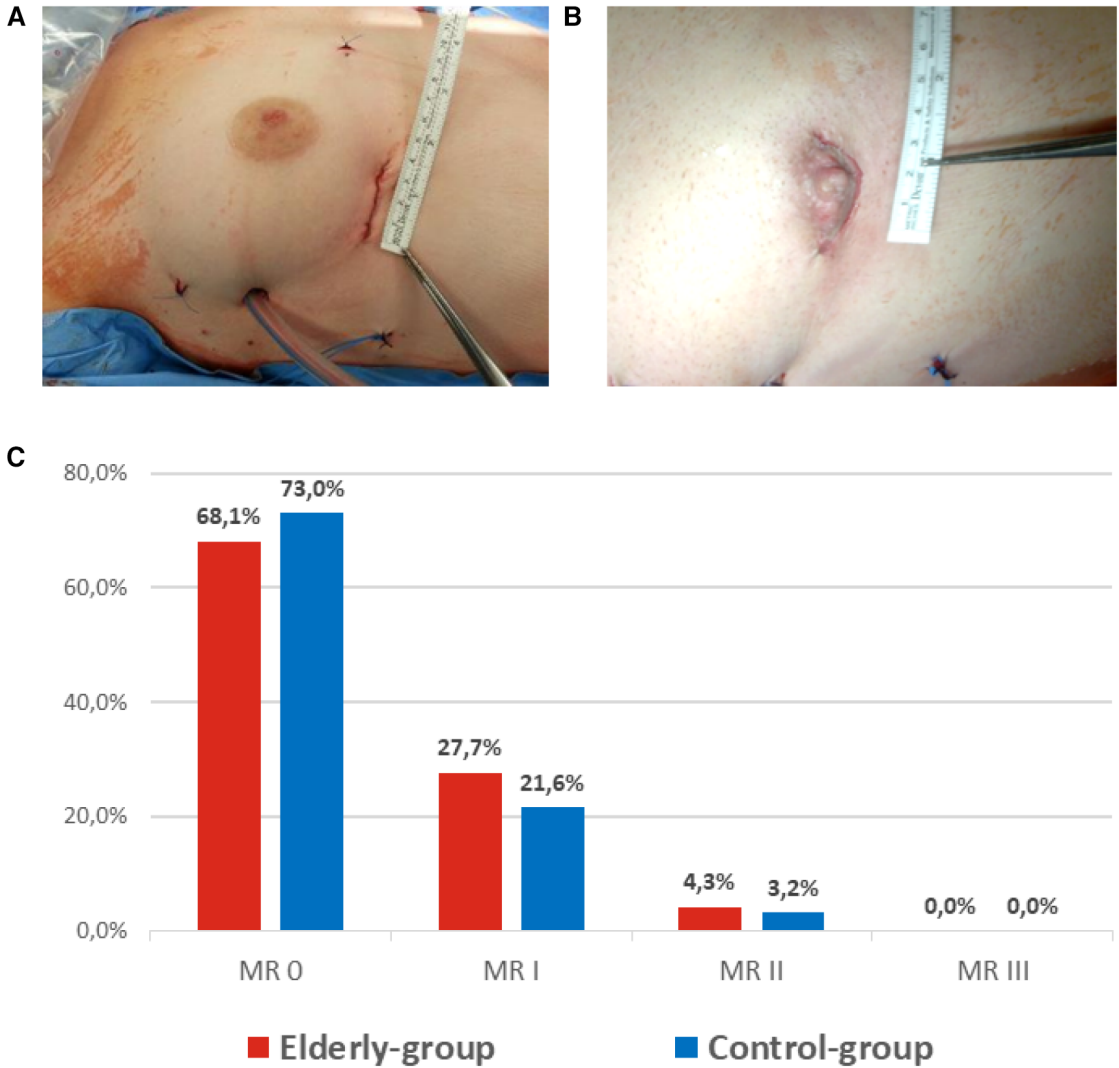
Cosmetic results (**A**, **B**) and perioperative outcome (**C**).

Median postoperative ventilation time was significantly increased in the elderly group [6.0 (4.6–9.3) h vs. 4.5 (3.0–6.3) h; *p* < 0.001]. Furthermore, length of stay on intensive care unit [2 (1–4) days vs. 2 (1–3) days; *p* < 0.001] and length of in-hospital stay [9 (7–13) days vs. 7 (6–9) days; *p* < 0.001] were longer in the elderly-group. Rates of perioperative myocardial infarction, low cardiac output syndrome, hemodialysis and surgical revision due to bleeding were similar (Table 3). Of note, incidence of perioperative stroke was similarly low (1 patient in each group; *p* = 0.224). At discharge, 68.1% vs. 73.0% of patients had no residual MR and 27.7% vs. 21.6% had mild residual MR (Figure 2C).

**Table 3:** Periprocedural outcome.

Variables	Elderly group (*n* = 91)	Control group (*n* = 665)	*p*-value
Postoperative ventilation time (hours), median (IQR)	6.0 (4.6–9.3)	4.5 (3.0–6.3)	<0.001
Revision due to bleeding, *n* (%)	9 (9.9)	56 (8.4)	0.639
Permanent pacemaker implantation, *n* (%)	6 (6.6)	17 (2.6)	0.027
Perioperative myocardial infarction, *n* (%)	0 (0.0)	1 (0.15)	1.0
Perioperative low-cardiac output, *n* (%)	5 (5.3)	25 (3.8)	0.393
Postoperative hemodialysis, *n* (%)	3 (3.3)	19 (2.9)	0.739
Perioperative stroke, *n* (%)	1 (1.1)	1 (0.15)	0.224
Length of stay on ICU (days), median (IQR)	2 (1–4)	2 (1–3)	<0.001
Length of postoperative in hospital stay (days), median (IQR)	9 (7–13)	7 (6–9)	<0.001
30-day mortality, *n* (%)	2 (2.2)	3 (0.45)	0.112

ICU, intensive care unit.

30-day mortality was 2.2% (2/91) vs. 0.45% (3/665 (*p* = 0.112). One patient in the elderly-group expired due to multi-organ failure 5 days after urgent MVS due to decompensated heart failure. A second patient committed suicide 16 days after surgery.

### MV re-operation and survival rates during follow up

**Figure 3:**
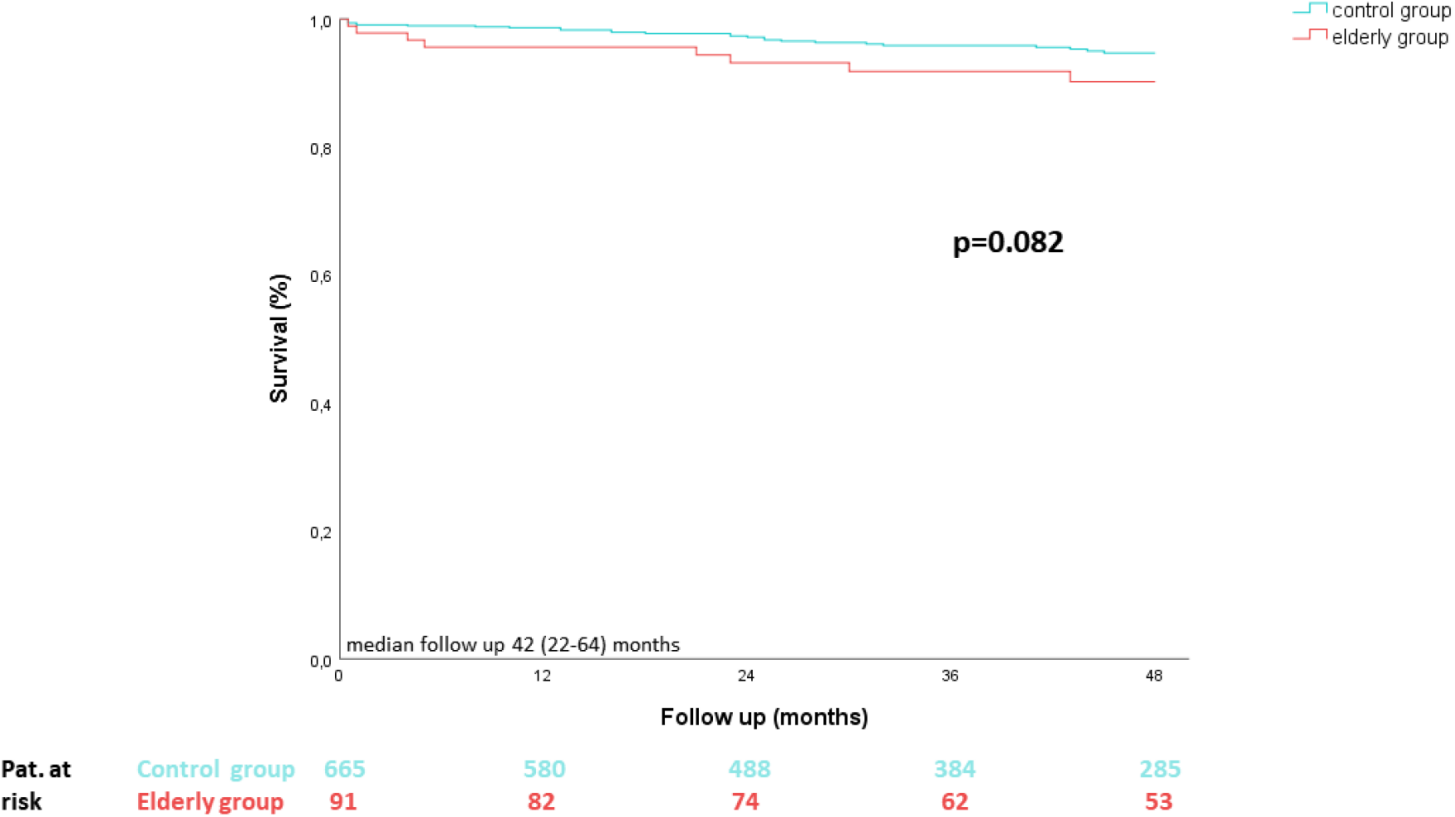
Kaplan–Meier survival analysis.

In contrast to the elderly group, in which MV re-operation was not necessary during a median follow up of 42 (22–64) months, 16 patients within the control group underwent MV re-operation (*p* = 0.135). Furthermore, Kaplan–Meier survival analysis revealed no statistical differences between groups (*p* = 0.082) (Figure 3).

## Discussion

Endoscopic MVS, which developed as an alternative to full-sternotomy, is traditionally performed in younger patients with low surgical risk. Nevertheless, in addition to transcatheter mitral valve repair strategies (e.g., TEER), minimally-invasive endoscopic MVS, may represent a potential therapeutic option for elderly patients (10).

In the present analysis, patients aged ≥75 years showed similarly low rates of perioperative complications compared to patients <75 years, despite increased prevalence of outcome-relevant comorbidities and overall increased surgical risk.

### Study population and preoperative patient characteristics

Patients ≥75 years had increased prevalence of comorbidities, impaired RV function and were more likely to present with NYHA class III symptoms and SMR in comparison to younger patients. Therefore, elderly patients exhibit an increased perioperative surgical risk (8), according to established risk stratification models (e.g., The Society of Thoracic Surgeons (STS) predicted risk of mortality (PROM) score) (STS PROM: 1.9% vs. 0.4%; *p* < 0.001) (Table 1) (11).. However, relevance of standard risk stratification tools to predict risk in elderly patients undergoing minimally-invasive MVS remains unclear (12). Therefore, in addition to risk score calculation, interdisciplinary heart-team discussion integrating patients' physical status, frailty, anatomical features, as well as life expectancy, is mandatory to achieve optimal patient outcome (13, 14). Of note, all patients included in the current analysis were assessed in a multidisciplinary team and defined as appropriate surgical candidates.

### Procedural characteristics, perioperative complications and mid-term follow up

Since introduction of minimally-invasive MVS (6), the technique evolved as an alternative to full-sternotomy, resulting in low rates of perioperative complications, reduced recovery time and superior cosmesis (Figures 2A,B) (7). Due to excellent 3D visualization (Figure 1B), endoscopic MVS can be routinely performed for treatment of a wide variety of mitral and tricuspid valve diseases (15). In addition to isolated MV leaflet prolapse, complex Barlow's disease (16), SMR due to ventricular dysfunction (17, 18), as well as infective endocarditis (19), can be targeted.

Repair rates within our series were 85.7% vs. 93.8% (*p* = 0.055). Of note, out of 13 patients within the elderly group, who underwent MV replacement, 3 patients had previously received TEER, 3 patients suffered from previous MV endocarditis with advanced destruction of leaflets, 4 patients had advanced leaflet degeneration with partial calcification and 3 patients showed SMR with severe mitral leaflet tethering. In accordance with current guidelines, in particular in the treatment of PMR MV repair was preferred over MV replacement (3), due to better short- and long-term survival, as well as fewer valve related complications, regardless of patients' age (20, 21). The preferred MV repair technique [e.g., “respect vs. resect” (22)], was performed with similar frequency in in both groups. As the implantation of pre-measured PTFE-loops (23) simplifies chordal replacement during minimally-invasive MVS, resulting in excellent long-term durability (24), it became the predominantly used technique (i.e., in 73.1% of all MV repair cases) at our institution.

Retrograde arterial perfusion due to cannulation of the femoral artery potentially represents a risk factor for perioperative stroke during minimally-invasive MVS, especially in patients with aortic or iliofemoral atherosclerosis, although conflicting data has been published (25). Direct cannulation of the right axillary artery to allow for antegrade arterial perfusion evolved as an alternative to conventional femoral cannulation during minimally-invasive MVS (26). Particularly in elderly patients with aortic atherosclerosis, antegrade perfusion may be associated with reduced perioperative stroke rates in comparison to retrograde perfusion (27) even though prospective randomized data does not exist. Of note, direct cannulation of the right axillary artery to facilitate antegrade arterial perfusion was performed in 27.5% of elderly patients in comparison to 4.2% of patients <75years (*p* < 0.001) and perioperative stroke rates were comparably low (1 patient in each group; *p* = 0.224) within our cohort.

Due to the absence of a control-group of patients treated with TEER or via full-sternotomy, the comparison to other MV treatment strategies is limited. Nevertheless, favorably low rates of perioperative myocardial infarction, low-cardiac output syndrome, hemodialysis and stroke within both groups, emphasize feasibility and safety of minimally-invasive MVS in elderly patients. Interestingly, perioperative complication rates seem to be lower than in recently published surgically treated elderly cohorts and comparable with real world TEER data (5). Of note, reduced postoperative ventilation time in the control group may reflect recent implementation of an enhanced recovery after cardiac surgery (ERAS) protocol, including patients <70 years at our institution. Expansion of the ERAS protocol from younger to elderly patients may potentially be of clinical value, as elderly patients may specifically benefit from rapid extubation, early postoperative mobilization and reduced duration of in-hospital stay (28).

Overall 30-day mortality was 0.7% (5/756), which compares favorably to published data (12). The numerical difference between groups (2.2% vs. 0.45%; *p* = 0.112), may be reflective of different baseline and procedural characteristics, e.g., higher rates of SMR and MV replacement in elderly patients (29).

In both groups, >95% of patients showed postoperative MR ≤ I at the time of discharge. Most importantly, freedom from MV re-operation was 100% during a median follow up of 42 months within the elderly group. Given the fact that residual MR > I, which is more frequently accepted after TEER in elderly patients (5), results in worse long-term outcome and survival (30), minimally-invasive MVS therefore represents a promising therapeutic option in elderly patients.

### Study limitations

Study limitations include the retrospective single-center study design. However, this is one of the largest consecutive series of elderly patients, undergoing endoscopic MVS at a specialized heart valve center, following institutional standards. Furthermore, despite differing age-related baseline characteristics within both groups, propensity score matching, or regression analysis was not included within our analysis. Therefore, the independent effect of age on the outcome after endoscopic MVS remains unknown. Due to the absence of a control-group of patients treated with TEER or via full-sternotomy, the comparison to other MV therapeutic strategies is limited. Nevertheless, the aim of our study was to demonstrate safety and feasibility of endoscopic MVS in elderly patients. Obviously, patients suffering from age-related contraindications for MVS, such as porcelain aorta or severe frailty, did not undergo MVS and consequentially were not included for analysis. Therefore, favorable perioperative results cannot generally be transferred to the elderly population. Nevertheless, results underline that age alone does not represent a contraindication for endoscopic MVS. Furthermore, it emphasizes the importance of interdisciplinary heart team assessment to select appropriate surgical candidates independent of age.

## Conclusion

Despite increased prevalence of outcome-relevant comorbidities and surgical risk, perioperative outcome of patients aged ≥75 years undergoing endoscopic MVS is favorable. Therefore, in addition to transcatheter approaches, endoscopic MVS is a valuable therapeutic option for selected elderly patients and should be taken in consideration during routine heart-team discussion.

## Data Availability

The raw data supporting the conclusions of this article will be made available by the authors, without undue reservation.

## Author's note

Data was presented at the DGTHG 52th Annual Meeting 2023 in Hamburg, Germany.
